# Supplementary material for: Parasitism of Hirsutella rhossiliensis on Different Nematodes and Its Endophytism Promoting Plant Growth and Resistance against Root-Knot Nematodes
Source: J Fungi (Basel). 2024 Jan 15;10(1):68. doi: 10.3390/jof10010068 (PMC10820206; doi:10.3390/jof10010068)
Supplement: Supplementary file 1 [file jof-10-00068-s001.zip › jof-2783228-supplementary.pdf]

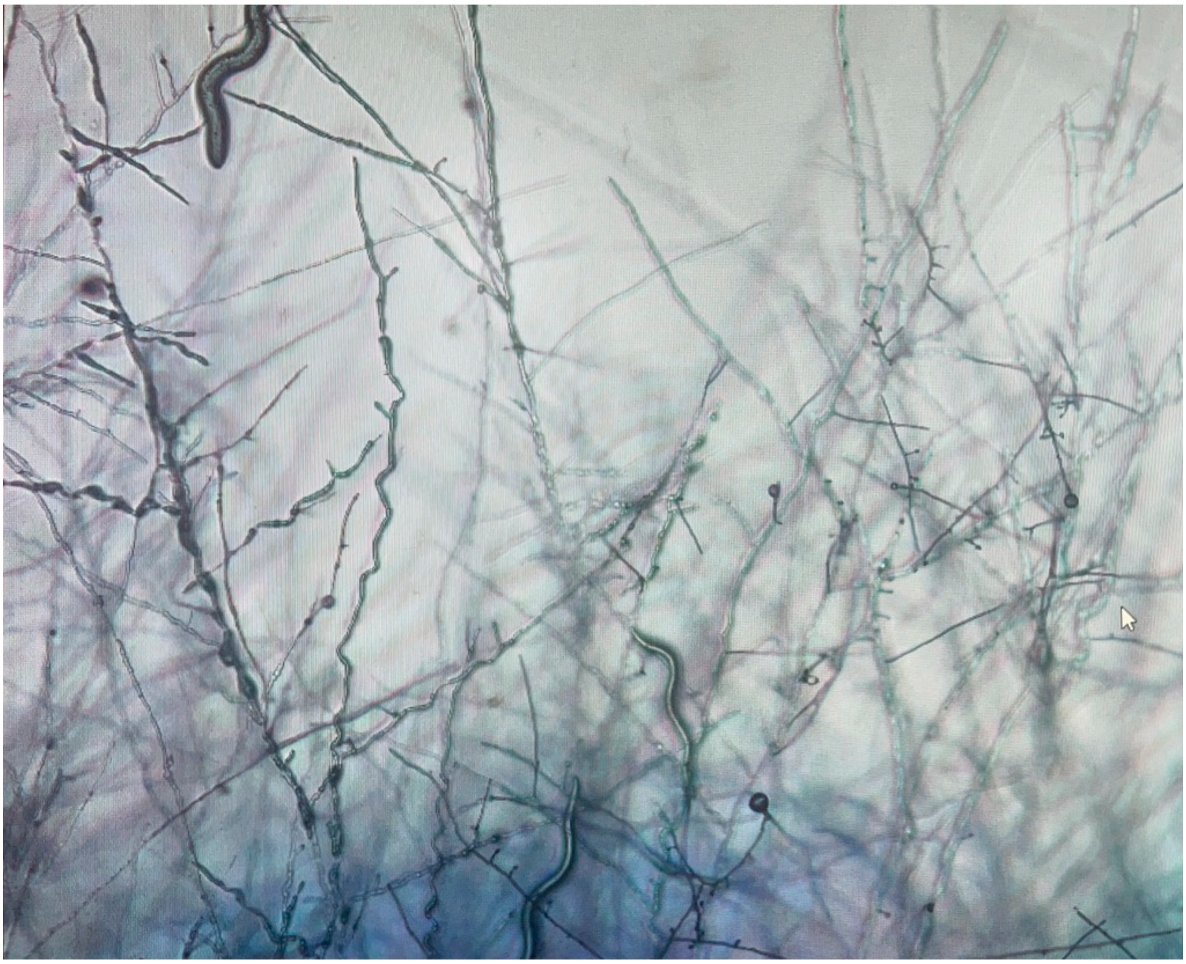

Figure S1. The root-knot nematode *M. incognita* on the fungal mat of *H. rhossiliensis* HR02. Image was taken under 10× objective lens and 10× ocular lens.

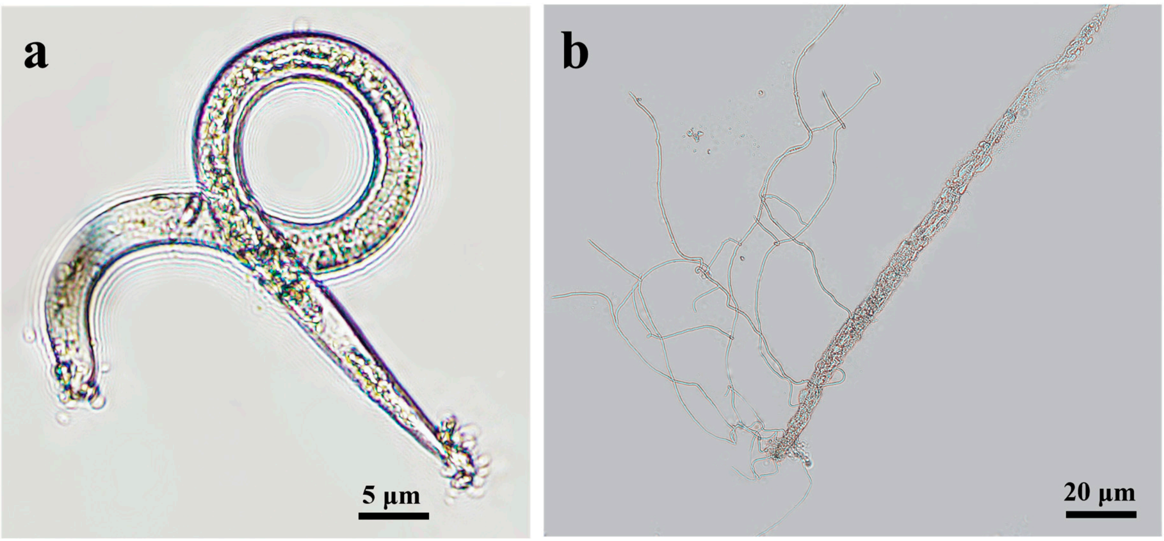

Figure S2. Infection of *H. rhossiliensis* HR02 on *M. incognita*. More conidia are gathered on the head and tail of the nematode (a) and hyphae grow out from the dead host (b).

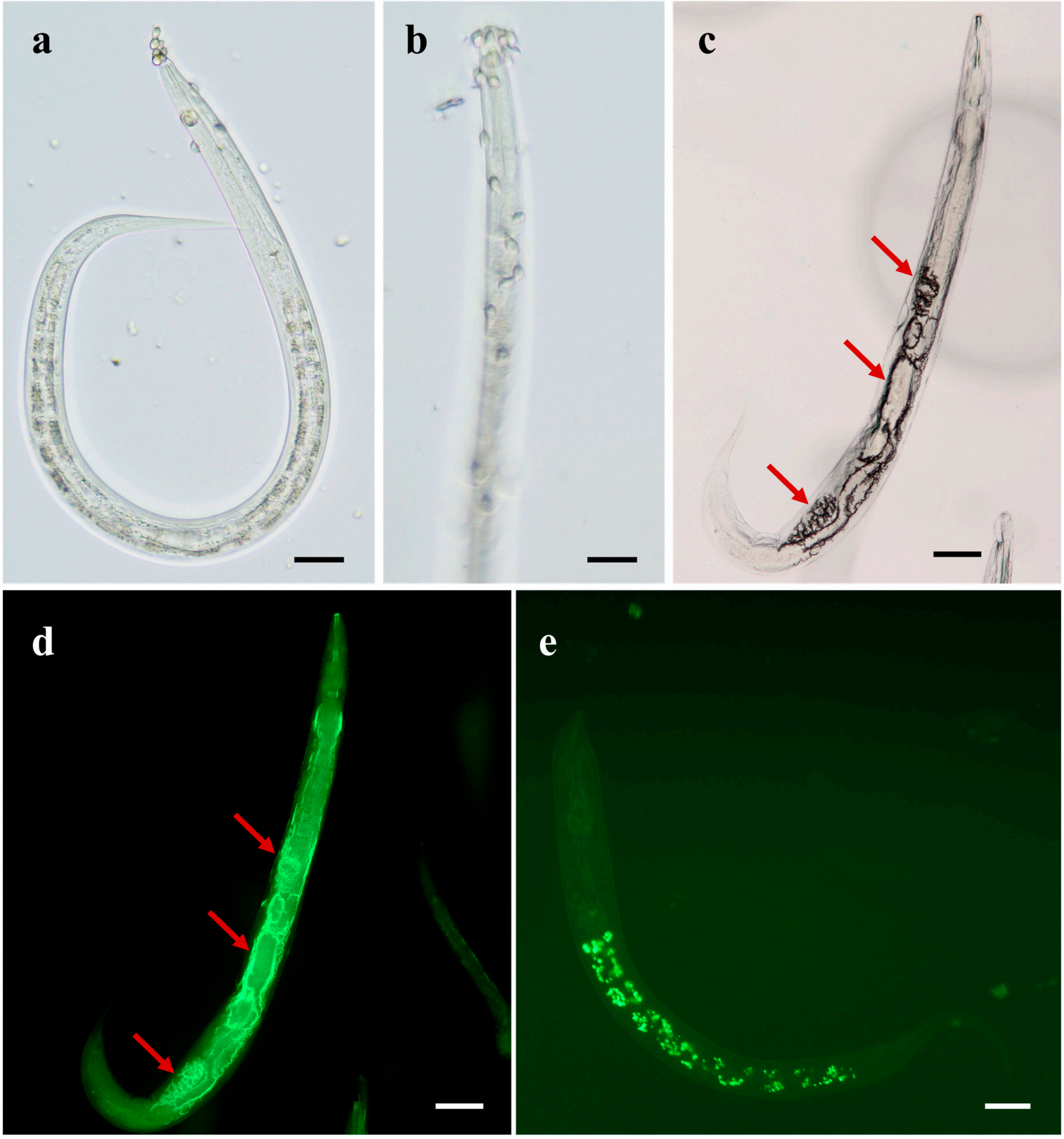

Figure S3. Infection of *H. rhossiliensis* HR02 on *C. elegans*. Conidia spores are attached to the cuticle (a); some enter into the intestine by swallowing from mouth (b); hyphae grow in the body (c); infected nematode under fluorescence condition (d); the control nematode with autofluorescence in the intestine (e).
